# Knowledge of pharmacists about anti-epileptic drugs in Sudan: a cross-sectional analytical study

**DOI:** 10.1186/s40780-025-00450-5

**Published:** 2025-05-20

**Authors:** Fatima O. Abdelsalam, Shahd A. Mohamed, Asgad A. Altrafi, Doha E. Balla, Abdalrahman M. Abdalrahman, Awab K. Atta, Abdalfattah M. Abdelrahim, Salsabil A. SeedAhmed, Alhumaira Wedaa, Yousif B. Hamadalneel

**Affiliations:** 1 Pharmacy Program, Wad Medani University of Science and Technology, Wad Medani, Sudan; 2 Department of Clinical Pharmacy and Pharmacy Practice, Faculty of Pharmacy, University of Gezira, Wad Medani, Sudan

**Keywords:** Epilepsy, Antiepileptic drug, Pharmacists, Sudan

## Abstract

**Introduction:**

Pharmacists play essential roles in the use of antiepileptic drugs (AEDs), inadequate knowledge among community and hospital pharmacists regarding AEDs poses a significant challenge in ensuring optimal medication management and patient care.

**Objective:**

This study aimed to assess the knowledge of pharmacists about antiepileptic drugs in Sudan.

**Method:**

This was a cross-sectional analytical study conducted in Atbara, Gadarif, Kassala, and Port Sudan, Sudan. Using stratified, convenience sampling technique. We collected data directly via a modified, validated English questionnaire from the 1st to the 30th of October 2024.

**Results:**

In total, 384 pharmacists were included. Among them, 300 (78.1%) were under 35 years of age, and the majority of participants were male 213 (55.5%), 157 (40.9%) had a basic bachelor’s degree, and 330 (85.9%) received specialized AEDs training. The overall median score of knowledge percentages among participants was 10, with an interquartile range of 30 {25– (-5)}. Only 25 participants (6.5%) demonstrated good knowledge, while a majority of 359 (93.5%) exhibited poor knowledge. There was a statistically significant correlation between years of experience (adjusted odds ratio (AOR) 0.437; 95% CI 0.224–0.855; p value 0.016) and training on AEDs (AOR 0.085; 95% CI 0.031–0.233; p value 0.001) with their level of good knowledge.

**Conclusion:**

Our study revealed that the majority of pharmacists in Sudan had a poor level of knowledge about AEDs. The level of knowledge was associated with years of experience and training on AEDs. These results highlight the need for pharmacists to have more training and more exposure to epilepsy and its management.

## Introduction

Epilepsy is a neurological disorder characterized by the occurrence of unprovoked seizures [1]. The World Health Organization estimates that 50 million people worldwide suffer from epilepsy and that 70% of those individuals might be seizure free with the right diagnosis and care [2]. Seizures fall into two main categories: generalized seizures and partial (focal) seizures [3]. One of the causes of epilepsy is excessive and aberrant neuronal activity [2], which is caused by either acquired or hereditary factors, including birth abnormalities, brain infections, brain tumors, and injuries to the brain [2].

The major objectives of treating epilepsy are to prevent seizures, minimize or avoid the negative effects of drugs, and avoid drug interactions [4]. The treatment of epilepsy includes medications known as antiepileptic drugs (AEDs), such as levetiracetam, phenytoin, carbamazepine, and valproate. It may also involve surgical procedures and non pharmacological approaches, such as vagus nerve stimulation, behavioral-modification therapies, diet, immunotherapy, and hormonal therapies [4]. First-line agents for status epilepticus include benzodiazepines such as midazolam, diazepam, and lorazepam [5].

Studies have shown that pharmacists are critical resources for patients with epilepsy seeking information about their condition and prescription drugs [3]. Enhancing patients’ knowledge and awareness may increase adherence and reduce drug interactions, which could improve clinical outcomes and enhance patients’ quality of life [3]. Community and hospital pharmacists evaluate the need for AEDs, prevent errors, and guide patients or their families [4, 6]. Medication management is the most often reported service provided by pharmacists to people with epilepsy, followed by education and counseling [7]. Few papers have focused on the use of care coordination documentation tools by doctors or pharmacists in the treatment of patients with epilepsy [6]. A study conducted in the United Arab Emirates reported that in addition to dispensing prescription drugs, community pharmacies frequently play a more active role in supporting individuals with epilepsy [6]. One of the main obstacles preventing pharmacists from giving patients the best care possible is a lack of knowledge and experience [4].

Inadequate knowledge among community pharmacists regarding AEDs poses a significant challenge in ensuring optimal medication management and patient care. A limited understanding of AEDs, including their mechanisms of action, side effects, drug interactions, and dosing regimens, may result in suboptimal therapy outcomes, medication errors, and compromised patient safety [8]. AEDs impact patients’ health since they frequently cause hepatotoxicity, renal stones, polycystic ovarian syndrome, aplastic anemia, rash, and a decrease in vital signs such as vitamins D and B12. However, 20–30% of patients treated with AEDs develop drug resistance, and up to half of patients experience side effects [9]. This underscores the essential role of pharmacists in providing medication management and regular monitoring to minimize adverse effects and improve treatment outcomes. Therefore, there is a critical need to evaluate pharmacist knowledge of AEDs to increase the knowledge and awareness of community pharmacists regarding AEDs, thereby improving the quality of pharmaceutical care provided to patients with epilepsy and ultimately optimizing treatment outcomes [10]. In Sudan, no research has been carried out to include Sudanese pharmacists from various states. Therefore, this study is the first of its type aimed to evaluate the knowledge of Sudanese pharmacists about AEDs and the factors that affect their knowledge in Port Sudan, Atbara, Gedaref, and Kassala, Sudan.

## Materials and methods

### Study design

A cross-sectional analytical study design.

### Study setting

The study was conducted in the cities of Atbara, Gedaref, Kassala, and Port Sudan, Sudan, from October 1 to 30, 2024. These cities are capital of the Nile River, Kassala, Gedaref, and Red Sea states. Given the ongoing conflict in Sudan, these locations were selected for their relative safety and the presence of a high number of pharmacists, ensuring a representative sample of community and hospital pharmacists throughout Sudan.

### Sample size

The sample size was calculated via Cochran’s Eq.  [11]:

N = (Z)^2[p*q]

e^2

*Z* = 1.96 *P* = 0.5 *Q* = 0.5 E = 0.05 Sample size: 384

### Participants

#### Inclusion criteria

All community and hospital pharmacists licensed by the Sudanese Medical Council in the mentioned cities were invited to participate in the research.

#### Exclusion criteria

Trainer or pharmacy students not licensed by the Sudanese Medical Council.

### Sampling technique

Non proportionally stratified and convenience sampling techniques were employed to select the participants. The sampling procedure was conducted in two steps. Initially, the study area was divided into four strata (Atbara, Gedaref, Kassala, and Port Sudan), i.e., probability stratified sampling. Second, 96 samples were collected from each stratum (non proportion) at several pharmacies and hospitals, i.e., nonprobability convenience sampling. The difficulty in formulating a sampling frame for all the pharmacists within each city made the probability sampling technique too difficult and the convenience sampling technique more appropriate in this study. The most significant issue with convenience sampling is that it may not represent the overall population. Therefore, we used multiple locations for sample collection. We visited different pharmacies and hospitals, met all the pharmacists presented at the time of the visit and invited them to answer the questionnaires at their workplace. After receiving their approval to participate in the study, we filled out the questionnaire. Depending on their availability, the questionnaire was completed at the time of the visit.

### Data collection

A modified and validated English questionnaire with two sections was used. With a focus on demographic/practice information and theoretical scenarios in epilepsy pharmacotherapy. Pharmacists chose responses to theoretical cases and provided the correct answer within 15 min without the use of an external source. Section one concerns demographic and practice information (nine questions), such as gender, age, degree and place from which the basic pharmacy degree was obtained, years of experience as community pharmacists/hospital pharmacists, specific training on AEDs during the pharmacy degree program and practice setting, and the number of people with epilepsy seen monthly. Section two includes 10 items (drawing a circle around the correct answer) related to the theoretical situation in the pharmacotherapy of epilepsy, including emergency situations in the pharmacotherapy of epilepsy, drug–drug interactions, the initial dose of lamotrigine, and the interpretation of the blood concentration of valproic acid. Pharmacists had to respond to each theoretical case by selecting one of the following multiple choices: (a) dispense; (b) dispense and recommend consulting a doctor upon the next appointment; (c) dispense after calling the doctor regarding drug–drug interaction (DDI); (d) do not dispense, call the doctor and refer the patient back to change the prescription; (e) do not dispense, call the doctor and urgently refer the patient to an emergency room; or (f) do not know [4, 8, 9].

We modified the questionnaire to better align with the actual situation of pharmacies and drugs in Sudan that are frequently used without change in the question objective. We modified four questions in section two: in question four we changed clarithromycin to Artemether + lumefantrine, question six changed lamotrigine to oral contraceptive, question eight changed tacrolimus to dextrose, and question 10 changed lamotrigine to meropenum.

### Questionnaire validity and reliability

To evaluate questionnaire validity and reliability, 40 pharmacists were asked to respond to the questionnaire. Reliability was assessed through Cronbach’s α, which indicated good internal consistency (α = 0.76), and a factor analysis value of > 0.4 indicated good reliability.

### Study variables

Dependent variable: Pharmacist knowledge about AEDs.

The independent variables include age, sex, pharmacy degree, place where the basic degree was obtained, years of experience, area of pharmacy, monthly epilepsy patient encounters, and specific training on AEDs.

### Data analysis

Data were collected, entered, and analyzed via the Statistical Package for the Social Sciences (SPSS) version 27. A normal distribution was determined via the Kolmogorov‒Smirnov test. Data that were normally distributed are expressed as mean +/- standard deviation, and not normally distributed are expressed as medians with interquartile ranges. The interquartile range (IQR) was calculated by subtracting Q1 from Q3 (Q3–Q1). After the applicability conditions were checked, the Pearson chi-square test and Fisher’s exact test were used to compare categorical data. Qualitative data are presented as frequencies (percentages). Univariable and multivariable binary logistic regression analyses were used to determine the factors associated with good pharmacist knowledge. All variables associated with pharmacist knowledge at a p value < 0.200 in the univariable model were included in the multivariable model via the enter method after being checked for multicollinearity and interaction. The results are displayed as odds ratios with 95% confidence intervals (CIs), and a statistically significant difference was considered at a p value ≤ 0.05. The knowledge levels were analyzed by assigning 1 point to each correct answer and zero points for the answer “I don’t know” or if the respondent did not answer the question. The pharmacist was informed in advance: to penalize guessing, 0.5 points well be deducted for each wrong answer to encourage the use “I don’t know” as a response to prevent point deductions, which effectively limits the potential for negative scoring and false positive results to reflect the actual knowledge level [10]. The total participant score is then divided by 10 and multiplied by 100 to obtain final scores as percentages {if participant achieved 5 out of 10 points = (5/10) x100 = 50%}, with a score of 50% or more taken as an indicator of good knowledge and a score of less than 50 as an indicator of poor knowledge [4, 8]. The overall knowledge percentage score was calculated as median with IQR.

### Ethical approval and informed consent

Ethical approval for the study was granted by the ethical committee, Ministry of Health in Kassala State, Sudan (7/10/2024). Written informed consent was obtained from each participant.

## Results

### Demographic and practice information

Our sample consisted of 384 participants divided equally into four areas. Among the pharmacists, the majority of the participants 213(55.5%) were male, and 300 (78.1%) were under 35 years. A total of 157 (40.9%) had a basic bachelor’s degree in a pharmacy (Table 1). Among the studied pharmacists, 246 (64.1%) had experience ranging from one to five years, and 330 (85.9%) had received specific training on AEDs during the pharmacy degree program and practice setting (Table 1).

**Table 1:** Characteristics of the study participants and scoring ≥ 50% on the 10-item test

Variable	*N* (%)	≥ 50% *N* (%)	< 50% *N* (%)	χ2 *p* Value
**Age (years)**				0.256
< 35	300 (78.1)	16 (64)	284 (79.1)	
36–40	66 (17.2)	7 (28)	59(16.4)	
≥ 40	18 (4.7)	2 (8)	16(4.5)	
**Gender**				0.233
Male	213 (55.5)	11 (44)	202 (56.3)	
Female	171 (44.5)	14 (56)	157 (43.7)	
**The place from where the basic pharmacy degree was obtained**				0.286
Governmental university	238 (62)	18 (72)	220 (61.3)	
Private university	146 (38)	7 (28)	139 (38.7)	
**Degree in pharmacy**				0.024
Bachelor of pharmacy	157 (40.9)	16 (64)	141 (39.3)	
Pharm.D	152 (39.6)	3 (12)	149 (41.5)	
Master of Science in Pharmacy	68 (17.7)	6 (24)	62 (17.3)	
Pharmacy PhD	7 (1.6)	0 (0)	7 (1.9)	
**Years of experience as community/hospital pharmacist**				0.071
< 1	72 (18.8)	2 (8)	70 (19.5)	
1– 5	246 (64.1)	14 (56)	232 (64.6)	
6– 10	60 (15.6)	8 (32)	52 (14.5)	
> 10	6 (1.6)	1 (4)	5 (1.4)	
**Type of pharmacist**				0.187
Community pharmacist	342 (89.1)	19 (76)	323 (90)	
Hospital pharmacist	42 (10.9)	6 (24)	36 (10)	
**Number of PWE seen monthly**				0.015
0	27 (7)	0 (0)	27 (7.5)	
1–10	169 (44)	18 (72)	151 (42.1)	
11–20	153 (39.8)	4 (16)	149 (41.5)	
> 20	35 (9.1)	3 (12)	32 (8.9)	
**Had training on AEDs during pharmacy degree program and practice setting**				0.001
Yes	330 (85.9)	12 (48)	318 (88.6)	
No	54 (14.1)	13 (52)	41 (11.4)	
**Where do you live?**				0.001
Port sudan	96 (25)	0 (0)	96 (26.7)	
Kassala	96 (25)	14 (56)	82 (22.8)	
Atbara	96 (25)	7 (28)	89 (24.8)	
Gedaref	96 (25)	4 (16)	92 (25.6)	

**Abbreviations**: AEDs: antiepileptic drugs, Pharm.D: Doctor of Pharmacy, PhD: Doctor of Philosophy, PWE: people with epilepsy

### Participants’ knowledge

For the 10-item questionnaire, the median score of knowledge percentages among participants was 10, with an interquartile range of 30 {25– (-5)}. Only 25 participants (6.5%) demonstrated good knowledge, while a majority of 359 (93.5%) exhibited poor knowledge.

Among the studied pharmacists, 71 (18.5%) correctly answered questions regarding the use of valproic acid during pregnancy (item #5). With respect to DDI, 161 (41.9%) of the pharmacists were familiar with the possibility of DDI of carbamazepine and artemether + lumefantrine (Coartem^R^) (item #4), 201 (52.3%) were familiar with valproic acid with a meropenum (item #10), and 75 (19.5%) were familiar with carbamazepine and oral contraceptives (item #6) (Table 2).

**Table 2:** Knowledge of pharmacists on issues related to antiepileptic drugs

		Response distribution					
		Dispense	Dispense and recommend to consult doctor upon next appointment	Dispense after calling the doctor regarding DDI	Do not dispense, call the doctor and refer the patient back to change the prescription	Do not dispense, call the doctor and urgently refer the patient to an emergency room	I do not know
**Questions**		N (%)	N (%)	N (%)	N (%)	N (%)	N (%)
1	A patient displays a prescription for lamotrigine 50 mg × 2/day. You see that the patient has a widespread red rash on their face and hands, and the patient reports that the rash has developed recently and its cause is unknown.	72 (18.8)	38 (9.9)	26 (6.8)	**60 (15.6)** ^*^	184 (47.9)*	4 (1.0)
2	A patient brings a new prescription for lamotrigine 100 mg × 2/day.	35 (9.1)	66 (17.2)	115(29.9)	**74 (19.3)**	84 (21.9)	10 (2.6)
3	A parent brings a prescription for levetiracetam 3000 mg/day for their 16-year-old Son — which he has been taking for over a month. The parent says the boy hears voices that order him to commit suicide.	23 (6.0)	46 (12.0)	118 (30.7)	65 (16.9)*	121 (31.5)*	11 (2.9)
4	A patient displays a chronic prescription for carbamazepine 400 mg × 2/day and a new temporary prescription for Artemether + lumefantrine (coartem^R^) 80/480 mg × 2 days for malaria.	38 (9.9)	41 (10.7)	56 (14.6)	**161 (41.9)**	78 (20.3)	10 (2.6)
5	A woman with epilepsy, regularly taking valproic acid 500 mg × 1/day determines that she is pregnant (unplanned, week 8). Would like to fill her prescription.	22 (5.7)	52 (13.5)	108 (28.1)	**71 (18.5)**	100 (26.0)	31 (8.1)
6	A.M. is a 23-year-old woman, She is in good health and regularly tacking carbamazepine 200 mg × 2/day and new prescription for oral Contraceptives	23 (6.0)	52 (13.5)	90 (23.4)	**75 (19.5)**	127 (33.1)	17 (4.4)
7	A patient has been taking valproic acid 500 mg × 2/day for half a year, and their lab test results show that the serum concentration of valproic acid is 110 mcg/mL (recommended concentration range: 50–100 mcg/mL). The patient feels well and wishes to fill the prescription.	40 (10.4)	**51 (13.3)**	38 (9.9)	204 (53.1)	33 (8.6)	18 (4.7)
8	A written prescription for phenytoin 100 mg and dextrose 5%, for the purpose of phenytoin dilution.	29 (7.6)	31 (8.1)	65 (16.9)	**150 (39.1)**	81 (21.1)	28 (7.3)
9	A mother to a 10-year-old boy says her son suffers from epileptic seizures once a month and is taking carbamazepine, valproic acid and topiramate. During the last night he experienced five seizures, and therefore she urgently requests to receive more carbamazepine from the pharmacist in order to give him an additional dose beyond normal daily dose (she has a prescription of carbamazepine 300 mg × 2/day for the next month).	20 (5.2)	26 (6.8)	45 (11.7)	115 (29.9)*	152 (39.6)*	26 (6.8)
10	A patient displays a chronic prescription for valproic acid 1000 mg/day and a new prescription for meropenum 1000 mg × 3/day.	32 (8.3)	32 (8.3)	45 (11.7)	**201 (52.3)**	53 (13.8)	21 (5.5)

**Abbreviations**: DDI: drug–drug interaction, correct answers are in bold, *both choices D) Do not dispense, call the doctor and refer the patient back to change the prescription and E) Do not dispense, call the doctor and urgently refer the patient to an emergency room were considered correct answers

### Factors associated with participant’s knowledge

The chi-square test and Fisher’s exact test were used to test the associations of demographic and practice factors with pharmacists’ knowledge scores, as shown in (Table 1). The results indicated that there was a statistically significant correlation between participants who scored ≥ 50% and those who scored < 50%, with the area where they lived (95% CI; p value < 0.001), the number of PWE they interacted with each month (95% CI; p value = 0.015), the degree of pharmacy (95% CI; p value 0.024) and specific training in AEDs (95% CI; p value < 0.001).

Binary logistic regression analysis was used to determine the exact strength between the demographics and the dependent variable and to investigate which factors were predictors of scoring ≥ 50% on the 10-item test. After checking of multicollinearity and interaction for all variables associated with pharmacist knowledge scoring ≥ 50% at a p value < 0.200 in the univariable model, age was excluded as it correlated with degree of pharmacy. Then, multivariable logistic regression was performed. There was a statistically significant correlation between community or hospital pharmacists and scoring ≥ 50% (adjusted odds ratio (AOR) 0.240; 95% CI 0.078–0.744; p value = 0.013) (Table 3), with 19 (76%) of those scoring ≥ 50% attributed to community pharmacists (Table 1). In addition, a statistically significant correlation was found between years of experience and scoring ≥ 50% (AOR 0.418; 95% CI 0.221–0.791; p value 0.007) (Table 3), with the highest percentages of 14 (56%) noted for those with 1–5 years of experience followed by 8 (32%) for 6–10 years of experience, and the lowest 1 (4%) for those with more than 10 years of experience (Table 1). Furthermore, there was a statistically significant correlation between training on AEDs and scoring ≥ 50% (AOR 0.068; 95% CI 0.024–0.196; p value 0.001) (Table 3), with the highest percentage of 13 (52%) noted for those did not received training on AEDs (Table 1).

**Table 3:** Univariable and multivariable binary logistic regression of factors associated with a score ≥ 50% on the 10-item test

Characteristic	COR (95%CI)	*p* value*	AOR (95%CI)	*p* value**
Gender	0.611 (0.270–1.382)	0.237		
Age	0.595 (0.320-1.104)	0.100		
Where do you live	0.918 (0.638 − 1.320)	0.644		
Are you community or hospital pharmacist	0.353 (0.132–0.941)	0.037	0.240 (0.078-0.744)	0.013
Years of experience	0.455 (0.250–830)	0.010	0.418 (0.221-0.791)	0.007
Degree in pharmacy	1.470 (0.834-2.592)	0.182	0.942 (0.520–1.706)	0.844
The place from where the basic pharmacy degree was obtained	1.625 (0.662 − 3.990)	0.290		
Had training on AEDs during pharmacy degree program and practice setting	0.119 (0.051–0.278)	< 0.001	0.068(0.024–0.196)	< 0.001
Number of PWE seen monthly	1.232 (0.716–2.117)	0.451		

**Abbreviations**: COR: crude odds ratio, CI: confidence interval, A0R: adjusted odds ratio, PWE: patient with epilepsy

## Discussion

This was a cross-sectional analytical study design aimed at assessing the knowledge of community and hospital pharmacists about AEDs in Atbara city, Gedaref city, Kassala city and Port Sudan city in Sudan, revealing significant insights into their understanding and potential implications for patient care. The majority of the pharmacists who participated in our study reported poor knowledge about AEDs; the responses to the clinical scenarios indicated a concerning level of uncertainty and inconsistency in practice. A limited understanding of AEDs, including their mechanisms of action, side effects, drug interactions, and dosing regimens, may result in suboptimal therapy outcomes, medication errors, and compromised patient safety.

In this study, the median score of knowledge percentage was 10, with an IQR of 30. This score is lower than that reported in another cross-sectional observational study conducted among community and hospitals pharmacists in Palestinian which reported a median score of 33.4, with an IQR of 33.3 [8], and lower than the median score reported in a descriptive cross-sectional study conducted among community pharmacists in Khartoum State, Sudan 36.68, with an IQR of 21 [4]. In addition, this finding was lower than that reported in a cross-sectional study conducted among community and hospital pharmacists in Israel, in which the authors calculated the mean, which was 48 [9]. This variation might be linked to the differences in study participants and study designs. This result highlights the urgent need to implement mandatory programs or training to increase pharmacists’ knowledge about AED.

In our study, only 6.5% of the respondents had good knowledge. Similar results were reported in a descriptive cross-sectional study conducted among community pharmacists in Khartoum State, Sudan, in which 14.7% of the participants had good knowledge [4]. Given these similarities, it is possible that the problem of poor knowledge is not unique to our sample but rather represents more general patterns in comparable groups or geographical areas. The result of this study was less than the observational cross-sectional study carried out among medical students, Sudan reported that 63% of students have a high level of knowledge, which can be explained by the fact that the academic environment may actively encourage students to update their knowledge [12]. Additionally, the findings of this study were less than those of most published studies, such as a cross-sectional study carried out among community pharmacists in the city of Ouagadougou in Burkina Faso, in which 48.21% of the respondents had good knowledge of epilepsy; a cross-sectional study conducted among community pharmacists in Libya reported that 60.0% had good knowledge about epilepsy and its treatment with AEDs [3]; and a cross-sectional observational study conducted among pharmacists with different backgrounds in Canada reported that 42.2% of the participants reported good knowledge of epilepsy management [13]. This clear variation between studies may be linked to the fact that the availability and quality of educational resources about epilepsy can vary widely across regions. Areas with better access to healthcare education and training programs may have higher levels of knowledge among the population [13]. Our analysis highlights a significant knowledge gap that necessitates further investigation into the reasons behind this ignorance and the integration of the results of previous research to create focused educational initiatives.

In this study, a notable result showed that no respondent’s pharmacist residing in Port Sudan achieved a score of 50% or higher, which is markedly different from other cities. This result suggests that there might be an underlying issue affecting the pharmacists in Port Sudan. Factors such as differences in the working environment, including workload might impact pharmacists’ ability to maintain or enhance their knowledge. Given that Port Sudan City now serves as the temporary administrative capital of Sudan due to the military conflict in the official capital, Khartoum. Additionally, economic conditions, specific health challenges and needs, and lack of access to continuing education, resources, or professional development opportunities could contribute to this low performance. Therefore, a more extensive assessment is needed to identify specific barriers faced by pharmacists in Port Sudan.

In our study, only 48.4% of the pharmacists recognized suicidal thoughts associated with levetiracetam. This is less than a cross-sectional study conducted among community and hospital pharmacists in Israel [9], in which 70% of the respondents recognized severe suicidal thoughts related to levetiracetam, and less than that documented in a descriptive cross-sectional study was conducted among community pharmacists in Khartoum State, Sudan, in which 68% of the respondents answered the question well [4]. Additionally, the findings of this study were less than those reported in a cross-sectional observational study was conducted among community and hospitals pharmacists in Palestine, where 79% of the pharmacists correctly answered the question [8]. Regional variations in pharmacy education and curricula may affect how well-versed pharmacists are in identifying and treating side effects, especially those that are as serious as suicidal thoughts. Pharmacists in countries with more comprehensive training on psychiatric medications may be more aware of potential side effects, including suicidal thoughts [6]. In many situations, recognizing suicidal thoughts linked to levetiracetam could save lives [10]. The relatively low identification rate of suicidal thoughts linked to levetiracetam among the pharmacists in this study highlights the importance of strengthening pharmaceutical education initiatives to guarantee that pharmacists are prepared to identify and handle medication-related mental health concerns.

In this study, 63.5% of the pharmacists knew that rash was a serious side effect of lamotrigine, which was in line with the findings reported in a descriptive cross-sectional study was conducted among community pharmacists in Khartoum State, Sudan (63.9%) [4], and in a cross-sectional observational study was conducted among community and hospitals pharmacists in Palestine 67% [8]. The finding of this study was higher than reported by a cross-sectional observational study conducted among pharmacists with different backgrounds in Canada, in which 20% of the participants were aware of the urgency of a lamotrigine rash and acknowledged the importance of seeking medical advice when dealing with rashes caused by lamotrigine [13], and a cross-sectional study conducted among community and hospital pharmacists in Israel reported that 20% of the pharmacists were aware of the urgency of a lamotrigine rash [9]. This difference in awareness raises issues regarding patient safety. If pharmacists lack awareness of the seriousness of lamotrigine rashes, it could affect their ability to provide effective patient counseling or identify possible drug-related concerns, which might result in delayed treatment or improper symptom management.

Approximately 42% of the participants could take the right action of dealing with DDI when carbamazepine was prescribed with artemether/lumefantrine (Coartem^R^). Carbamazepine induces hepatic enzymes, which can lead to decreased levels of artemether/lumefantrine in the body and potentially reduce the effectiveness of Coartem^R^ [14]. Owing to the prevalence of malaria in Sudan and the fact that artemether/lumefantrine (Coartem^R^) is the first line of malaria treatment, we need to be more aware that Coartem^R^ should not be prescribed with carbamazepine. Therefore, it is important to consult a healthcare professional before dispensing these medications to ensure safe and effective drug use. Additionally, regarding the DDI of carbamazepine, only 19.5% of our pharmacists understood the interaction between carbamazepine and oral contraceptives. Carbamazepine induces liver enzymes, which are responsible for the metabolism of hormonal contraceptives and result in decreased serum levels of contraceptive hormones and an increased risk of contraceptive failure, leading to unintended pregnancy [15].

With respect to valproic acid DDI, 52.3% of our pharmacists took the right action when valproic acid was prescribed with the meropenum. A retrospective study of patients treated with valproate medications revealed that the analysis of the drug interaction between valproic acid and meropenem indicated an average decrease in valproic acid levels of 77% (range, 34 to 99%) and aggravated epileptic seizures in 48% of the patients [16]. These results emphasize the importance of pharmacist involvement in handling DDIs and the wide consequences for patient safety and the effectiveness of therapy. Continuous training for pharmacists about DDIs and their possible effects is crucial for enhancing care for patients taking various medications. On the other hand, a cross-sectional study conducted in Khartoum state, Sudan, to evaluate valproic acid drug interactions revealed that 43.8% of respondents selected the correct action regarding the interaction of valproic acid with lamotrigine [5]. One of the main areas where a skilled pharmacist could be helpful is in screening for and warning about DDIs. The percentage of DDIs may increase as a result of pharmacists’ inadequate understanding of drug interactions related to epilepsy. Therefore, pharmacists need to easily access the most recent clinical guidelines, drug interaction databases, or pertinent literature that can assist them in screening DDIs.

In terms of AED safety, in this study, only 18.5% of the participants knew that valproic acid taken during pregnancy is associated with a greater risk of birth defects, which is less than that reported in a descriptive cross-sectional study was conducted among community pharmacists in Khartoum State, Sudan, which revealed that 42.4% of participants do not dispense, call the doctor and refer the patient back to change the prescription [4], and a cross-sectional observational study was conducted among community and hospitals pharmacists in Palestine revealed that 56.6% of pharmacists do not dispense, call the doctor and refer the patient back to change the prescription [8]. The small proportions of pharmacists who send patients back to doctors or who refrain from dispensing the medication raise concerns about patient safety. This emphasizes the importance of improved communication among patients, pharmacists, and doctors who prescribe medication. Pharmacists are essential in managing medications and should be promoted to serve as advocates for patient safety.

With respect to factors associated with the knowledge of pharmacists, multivariable logistic regression revealed that there was a statistical significant correlation between community or hospital pharmacist and scoring ≥ 50%, with (76%) of those scoring ≥ 50 attributed to community pharmacists. Although the hospital pharmacists generally are more likely to have good knowledge than the community pharmacists, this difference can be attributed to the reality that AEDs in Sudan are dispensed primarily in community pharmacies, leading to greater exposure for community pharmacists to these medications in comparison to hospital pharmacists. In addition, a statistically significant correlation was found between years of experience and scoring ≥ 50%, with the highest percentage of (56%) noted for those with 1–5 years of experiences and the lowest (4%) for those with more than 10 years of experience. This finding might appear illogical, but it might indicate newer education, updated curricula, or a reduced dependence on routine practice without ongoing education, in addition to that younger pharmacists are increasingly utilizing modern digital tools and platforms, including mobile drug reference applications (e.g., Up-to-date, Medscape), artificial inelegance-driven clinical decision support systems, and online continuing education courses to broaden their knowledge. On the other hand, more experienced pharmacists may lack recent education updates, resulting in knowledge gaps. Furthermore, there was a statistically significant correlation between training on AEDs and scoring ≥ 50%, with the highest percentage of (52%) noted for those did not received training on AEDs. While training is typically thought to enhance knowledge, this data shows that training is linked to low knowledge scores. This discordances can be explained by the individuals lacking training may depend on recent personal learning updates or self-education. On the other hand, the effectiveness and substance of training programs could be inadequate. Therefore, further research should be implemented particularly to answer why trained pharmacists have low knowledge compared to untrained pharmacist. The result of this study was inconsistent with that presented in a cross-sectional study conducted among community and hospitals pharmacists in Palestine, multiple logistic regressions revealed that training on epilepsy and AEDs more likely to achieved ≥ 50% than those who did not receive training on AEDs [8], and in descriptive cross-sectional study was conducted among community pharmacists in Khartoum State, Sudan, which revealed that having a higher degree in pharmacy (MSc or PhD) is the only factor that was significantly associated with good knowledge, with scores ≥ 50% on the 10-item test [4]. These results highlight the importance of continuous professional development efforts and specific training programs to improve pharmacists’ understanding of AEDs. It is crucial to address any educational and training deficiencies in different practice environments to ensure that pharmacists are properly prepared to manage patients effectively in this field. Moreover, future research could further investigate the causes of these differences and assess how various training methods affect knowledge retention and its application in practice.

### Study strengths and limitations

The strengths of this study include the following: This study covered four states in Sudan, making it more representative of Sudanese pharmacies. We did not allow pharmacists to use any resources to find answers to the survey questions in this study. The limitations of this study include the following: the use of a cross-sectional study design represents one point in time and restricts the ability to track changes in knowledge over time; the use of convenience sampling may not represent the overall population; and self-reporting can result in biased responses for a number of reasons, such as social desirability bias, where people give answers that they think are more positive or acceptable than their actual actions.

## Conclusion

This study revealed that the majority of pharmacists in Sudan had a poor level of knowledge about AEDs. The level of knowledge was associated with years of experience, whether the respondent was a community or hospital pharmacist, and training on antiepileptic drugs. The results of this study highlight the need for pharmacists to have more training and more exposure to epilepsy and its management. Therefore, we recommend [1] providing and executing extensive training courses for pharmacists that are geared toward AEDs and appropriate for both community and hospital pharmacies; [2] including courses concerning AEDs in continuing education and professional development requirements for pharmacists; [3] encouraging pharmacists to take part in online courses, workshops, and seminars to stay current in new advancements; and [4] carrying out longitudinal research to evaluate pharmacists’ knowledge of AEDs, identify gaps, and assess the effectiveness of interventions over time.

## Data Availability

The datasets used and/or analyzed during the current study are available from the corresponding author upon reasonable request.
